# “Green Is the Color”: An Update on Ecofriendly Aspects of Organoselenium Chemistry

**DOI:** 10.3390/molecules27051597

**Published:** 2022-02-28

**Authors:** Juliano B. Azeredo, Filipe Penteado, Vanessa Nascimento, Luca Sancineto, Antonio L. Braga, Eder João Lenardao, Claudio Santi

**Affiliations:** 1 Programa de Pós-Graduação em Ciências Farmacêuticas, Universidade Federal do Pampa, Uruguaiana, Uruguaiana 97501-970, RS, Brazil; julianoazeredo@unipampa.edu.br; 2 Laboratório de Síntese Orgânica Limpa-LaSOL-CCQFA, Universidade Federal de Pelotas-UFPel, P.O. Box 354, Pelotas 96010-900, RS, Brazil; penteado.filipe@gmail.com (F.P.); lenardao@ufpel.edu.br (E.J.L.); 3 Laboratório SupraSelen, Departamento de Química Orgânica, Instituto de Química, Campus do Valonguinho, Universidade Federal Fluminense, Niteroi 24020-150, RJ, Brazil; 4 Group of Catalysis Synthesis and Organic Green Chemistry, Department of Pharmaceutical Sciences, University of Perugia, Via del Liceo 1, 06100 Perugia, Italy; luca.sancineto@unipg.it; 5 Departamento de Química, Universidade Federal de Santa Catarina—UFSC, Florianopolis 88040-900, SC, Brazil; braga.antonio@ufsc.br

**Keywords:** selenium, catalysis, green chemistry, bioinspired catalysis, flow chemistry

## Abstract

Organoselenium compounds have been successfully applied in biological, medicinal and material sciences, as well as a powerful tool for modern organic synthesis, attracting the attention of the scientific community. This great success is mainly due to the breaking of paradigm demonstrated by innumerous works, that the selenium compounds were toxic and would have a potential impact on the environment. In this update review, we highlight the relevance of these compounds in several fields of research as well as the possibility to synthesize them through more environmentally sustainable methodologies, involving catalytic processes, flow chemistry, electrosynthesis, as well as by the use of alternative energy sources, including mechanochemical, photochemistry, sonochemical and microwave irradiation.

## 1. Introduction

Since the second half of the last century, organoselenium compounds have emerged as a useful building block in organic synthesis, being employed in the form of radicals, electrophiles and nucleophiles for the construction of a wide range of chemical bonds, which confirms their importance in the modern synthetic chemistry [1].

Furthermore, driven by the discoveries about the role of selenoproteins as an important class of mammalian antioxidant enzymes, the biological activities of small molecules organoselenium derivatives have been extensively investigated, evidencing the biological potential of this class of compounds [2,3,4].

**Figure 1:**
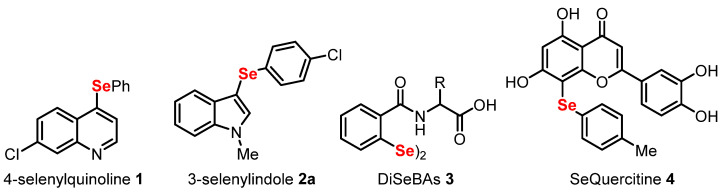
Bioactive organoselenium compounds.

Some recent impressive examples can be summarized by 4-selenylquinoline **1** [5]; a powerful anti-inflammatory compound when compared to the worldwide marketized Meloxicam^®^, and 3-selenylindole **2** [6,7], which has been demonstrating promising results against mood and neurodegenerative diseases; worthy of be mention are the DiSelenobisBenzAmides (DiSeBAs, **3**) endowed with potent and broad anti-HIV activity [8] due to their ability to inhibit NCp7 [9,10] and Se-Quercetines (**4** is the prototypical compound (Figure 1), capable of blocking the replication of SARS-CoV-2 [11]. For this reason, different synthetic approaches to accessing organoselenium compounds are continuously advancing, with ingenious methodologies being constantly reported in the literature.

The application of Green Chemistry concepts in synthetic chemistry has redesigned the paradigms, opening **up** new horizons and setting trends for the development of new approaches to the construction of important chemical bonds [12,13]. In this context, the application of principle #6 (Design for Energy Efficiency) has been proving to be important to circumvent the use of classical heating sources (thermal heating) by employing energy sources with low energy demand, including electromagnetic wave sources (microwaves and visible light), acoustic waves (sonochemistry), electrolytic cells (electrosynthesis) and mechanical energy (mechanochemistry). Among the main advantages, reactions control and selectivity are remarkably increased in comparison to classical thermal approaches, once the required reaction conditions are generally smooth [13,14].

Selenium chemistry, with particular emphasis on catalysis, was proposed for the first time as a powerful and potentially green alternative in 2009 [15], and five years later the advances in the field were collected in a widely cited review article [16]. Thus, in this review, we intend to cover the recent relevant advances in the synthesis of organoselenium compounds using alternative energy sources. Selected examples were chosen, aiming to illustrate some currently attractive trends for readers, with a separate discussion of some specific synthetic improvements obtained from the use of microwave-assisted, sonochemical, electrochemical, mechanochemical and visible light reaction conditions. Furthermore, important achievements for the synthesis of organoselenium compounds under continuous flow conditions are also reported.

## 2. Application of Non-Conventional Energy Sources in Organoselenium Chemistry

### 2.1. Microwave-Assisted Reactions

Microwave irradiation in organic synthesis is an important tool that has been used with the purpose of preparing several classes of compounds [17,18,19,20]. Microwave-accelerated reactions generally occur faster when compared to those with conventional heating, which guarantees high energy efficiency for these methods. This is because microwave irradiation comes into direct contact with the reactant molecules, without the need for prior external heating. This causes the activation energy to be reached faster and consequently the desired products are obtained more quickly. The speed at which these reactions occur also promotes a reduction in the formation of undesirable side-products, which facilitates the purification steps [21].

**Scheme 1:**
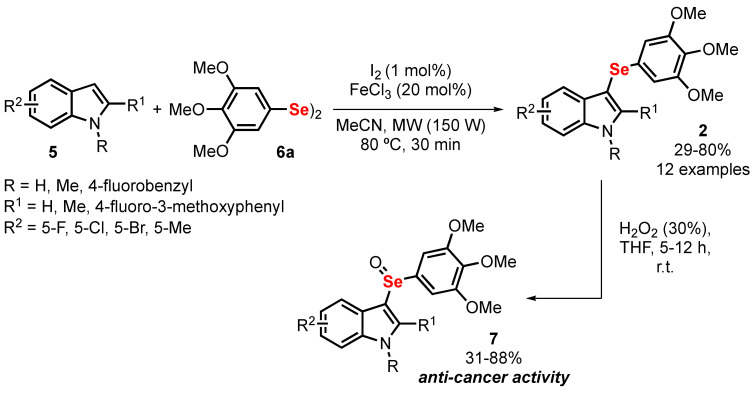
MW-promoted synthesis of 3-selenylindoles (**2**).

Regarding the synthesis of organochalcogen compounds, microwave irradiation has already been successfully applied to the functionalization of many organic substrates [22,23]. In this context, Wen and coworkers used MW to accelerate the preparation of 3-selenyl-indoles **2 [24]**. The desired products were achieved in only 30 min through the reaction of indoles **5** with 1,2-bis-(3,4,5-trimethoxyphenyl)diselenide **6a** in the presence of FeCl_3_ and iodine as catalyst in up to 80% yield. (Scheme 1). The prepared selenylindoles **2** and the corresponding selenoxide derivatives **7** have shown to be promising molecules with respect to their antiproliferative activity against three human cancer cell lines.

**Scheme 2:**
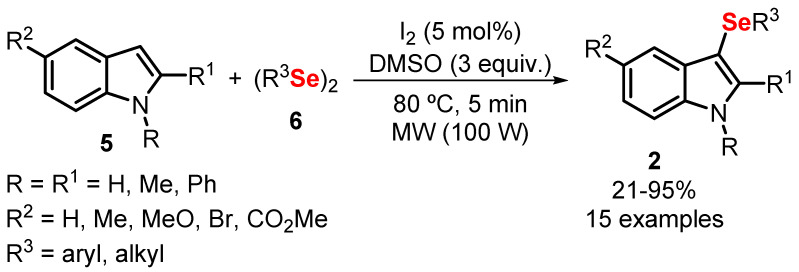
Microwave-assisted synthesis of 3-selenylindoles **2**.

Braga and co-workers described the direct selenylation of the indoles **5** using diorganoyl diselenides **6** mediated by catalytic iodine and three molar equivalents of DMSO in a solvent- and metal-free reaction medium. The protocol allowed the preparation of a series of 3-selenylindoles **2** in only 5 min under microwave irradiation in up to 95% yield (Scheme 2). The scope of this method was extended to the preparation of 3-arylsulfenylindoles [25].

**Scheme 3:**
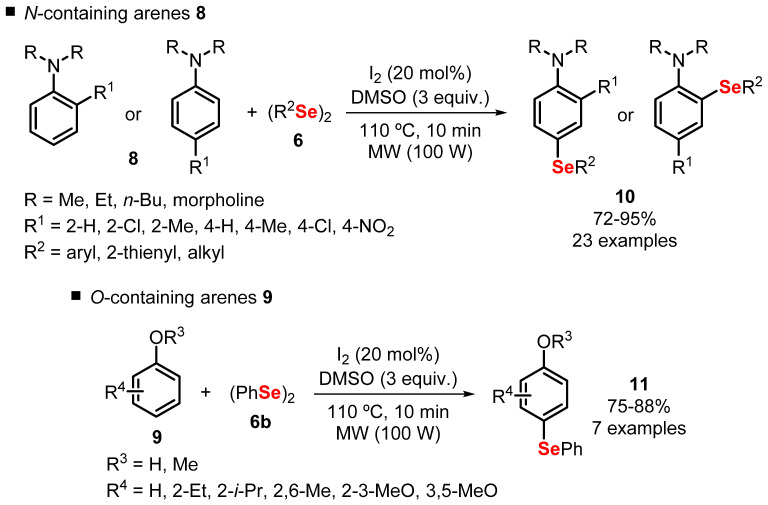
Microwave-assisted selenylation of *N*- and *O*-containing arenes **8** and **9**.

Later on, the same group extended the developed protocol for the direct selenylation of activated *N*- and *O*-containing arenes **8** and **9** (Scheme 3). The unsymmetrical selenides **10** and **11** were obtained in high yields, using 20 mol% of iodine and three equivalents of DMSO after 10 min under MW irradiation. This method was also extended to the functionalization of *O*-containing arenes [26].

**Scheme 4:**
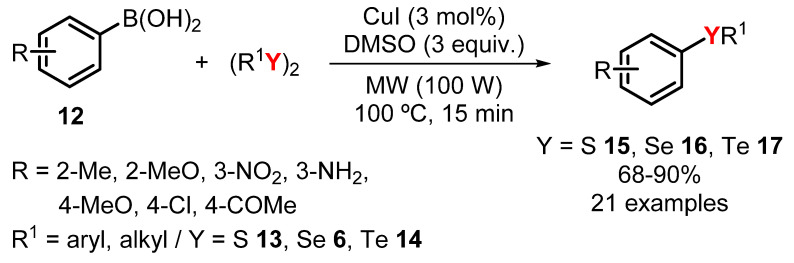
MW-driven synthesis of unsymmetrical chalcogenides **15**, **16** and **17**.

Microwave was also employed in the copper-catalyzed cross-coupling reaction between aryl boronic acids **12** and diorganoyl dichalcogenides **13**, **6** and **14**. In the presence of 3 mol% of CuI and three equivalents of DMSO, a series of unsymmetrical sulfides **15**, selenides **16** and tellurides **17** were obtained in only 15 min and in yields that ranged from 68% to 90% (Scheme 4) [27].

**Scheme 5:**
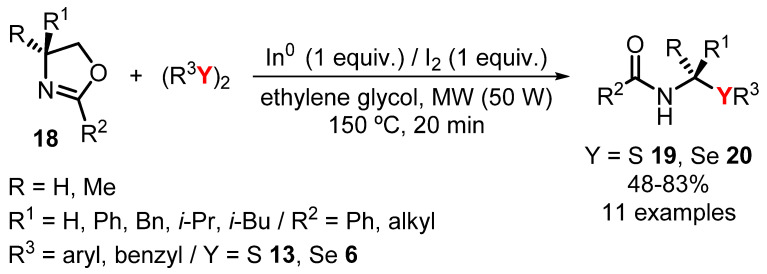
Microwave-assisted synthesis of β-chalcogenamides **19** and **20**.

In 2020, Galetto and co-workers reported the microwave-driven regioselective ring opening of 2-oxazolines **18** in the presence of diorganoyl diselenides **6**, one equivalent of indium and one equivalent of iodine. This protocol allowed the preparation of a series of structural diverse β-selenamides **20** in 20 min with up to 83% yield (Scheme 5) [28]. The methodology was also extended to the preparation of β-sulfuramides **19**, employing diorganyl disulfides **13** as substrate.

**Scheme 6:**
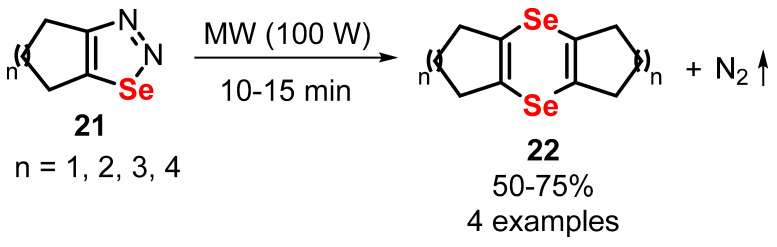
MW-assisted synthesis of **22**.

The microwave-assisted dimerization of 1,2,3-selenadiazoles **21** was reported by Khanna and co-workers. With this method, four bis(cycloalkeno)-1,4-diselenins **22** were obtained in times that ranged from 10 to 15 min (Scheme 6). The authors also reported the application of the 1,4-diselenins in nanotechnology as source of selenium for the synthesis of Cd selenium quantum dots (QDs) [29].

### 2.2. Sonochemistry

During the last decades, ultrasound irradiation has become an interesting alternative to fossil-based energy sources in the context of synthetic organic chemistry [30], promoting several types of reactions efficiently. Due to the highly efficient energy transfer, based on the acoustic cavitation effect, the low requirement of energy allows the realization of several transformations in short reaction times [31].

**Scheme 7:**
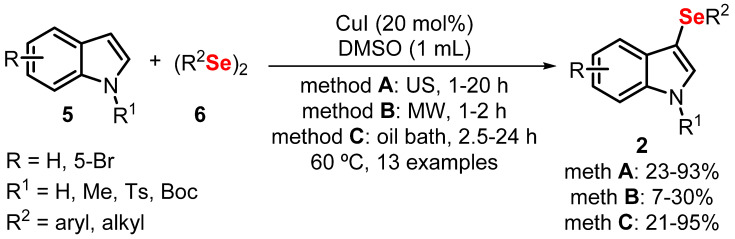
Preparation of 3-selenylindoles **2** via US, MW or conventional heating.

In recent years, the application of ultrasound irradiation to construct organoselenium compounds has been opening up new frontiers, affording more efficient processes and allying short reaction times and high yields [32]. In this context, some recent remarkable examples can exemplify specific trends. In 2015, Lenardão and co-workers reported an ultrasound-mediated Cu(I)-catalyzed electrophilic selenylation of indoles **5**, by using diorganyl diselenides **6**, accessing 3-selenylindoles **2** in poor to excellent yields [33]. In order to compare the efficiency of ultrasound irradiation (US) with different energy sources, the authors carried out a study of the reaction scope by employing ultrasound and microwave irradiation (MW), as well as conventional heating (oil bath) (Scheme 7).

In general, the results demonstrated that ultrasound irradiation and conventional heating were efficient in converting the starting materials to the respective products **2**, while microwave irradiation was not appropriate to promote the transformation. Among them (US and oil bath), in general, ultrasound irradiation was more efficient, giving the desired products **2** in a shorter reaction time. Additionally, excellent substituent toleration was observed, and several electron-rich and -deficient substrates were smoothly employed as reaction partners (Scheme 7).

**Scheme 8:**
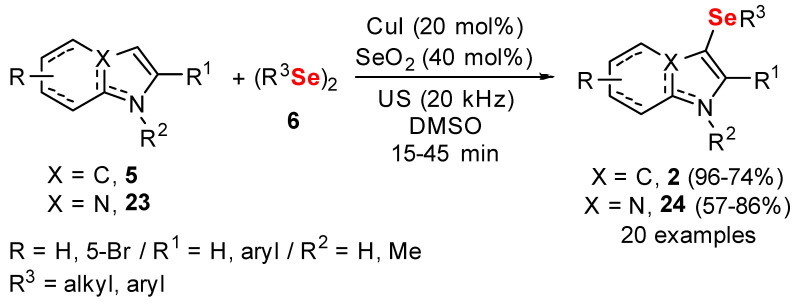
SeO_2_-activated synthesis of selenium containing heteroaromatics **2** and **24**.

Two years later, Lenardão and co-workers reported an updating study, affording a more efficient ultrasound-mediated Cu(I)-catalyzed protocol to access 3-selenylindoles **2** by employing SeO_2_ (40 mol%) as additive [34]. The addition of SeO_2_ as a co-oxidant species led to a remarkable improvement, greatly reducing the reaction times from hours to a few minutes and enhancing the product yields. In addition, authors expanded the reaction scope, employing the imidazo[1,2-*a*]pyridines **23** as substrate and accessing the 3-(organylselanyl)imidazo[1,2-*a*]pyridines **24** in moderate to good yields (Scheme 8).

**Scheme 9:**
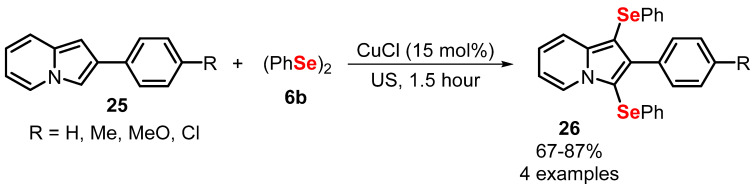
US-promoted synthesis of bis-selenylindolizines **26**.

More recently, the authors have expanded the method for the synthesis of 1,3-bis(phenylselenyl)indolizines **26** through an ultrasound-promoted Cu(I)-catalyzed reaction between 2-phenylindolizine derivatives **25** and diphenyl diselenide **6b** [35]. The reactions were conducted smoothly for 1.5 h in the presence of CuCl (15 mol%) to afford the desired products **26** in moderate to very good yields (Scheme 9).

**Scheme 10:**
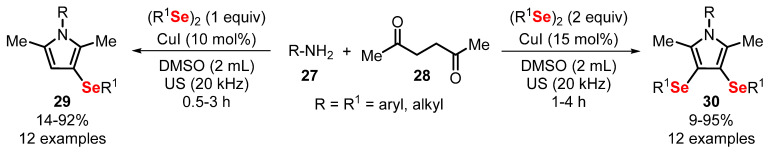
Synthesis of selenylpirroles **27** and **28**.

Alves, Perin and co-workers also explored the synergism between Cu(I) species and ultrasound irradiation in the catalytic selenylation of heteroaromatic systems, reporting, in 2019, a simple and efficient multicomponent protocol for the selective synthesis of *N*-substituted mono- and bis-selenylpyrroles **29** and **30**, by reacting primary amines **27**, 2,5-hexanediones **28** and diorganyl diselenides **6** [36]. Under the optimized reaction conditions the desired products were obtained in poor to excellent yields. In general, the products **29** and **30** were obtained in a very similar yield range, although the formation of the product **30** required long reaction times (Scheme 10).

**Scheme 11:**
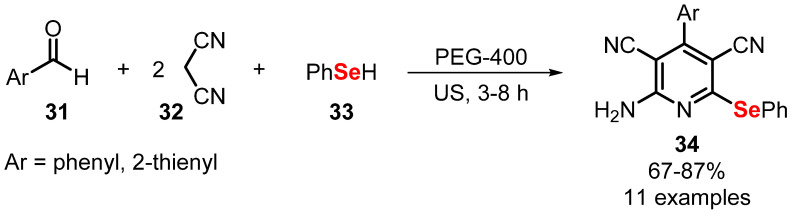
US-activated synthesis of selenylpyridines **34**.

In 2015, Choudhury and co-workers developed an ultrasound-promoted synthesis of highly functionalized selenylpyridines **34** in the presence of PEG-400 as solvent through the multicomponent reaction of aldehydes **31**, malononitrile **32** and benzeneselenol **33**, with the simultaneous formation of four new bonds (two C–C, one C–N and one C–Se), presenting an excellent electronic group tolerance [37] (Scheme 11).

**Scheme 12:**
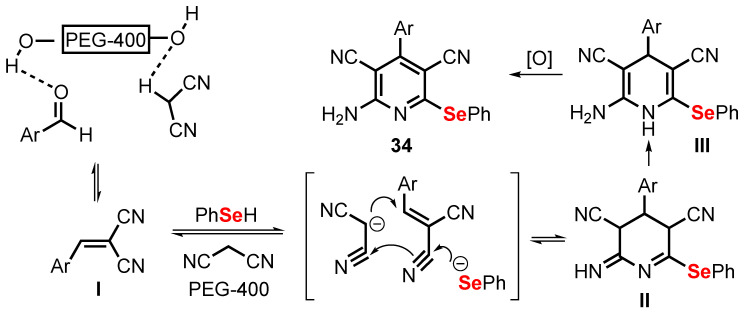
Reaction mechanism for the formation of product **34**.

The proposed reaction mechanism starts with a Knoevenagel condensation step in the presence of PEG-400 to form the yilidine intermediate **I**. Then, the intermediate **I** undergoes a simultaneous nucleophilic attack by the benzeneselenol **33** and by the malononitrile **32**, triggering an intramolecular cyclization to provide the intermediate **II**, which tautomerizes to afford the 1,4-dihydroselenopyridine **III**. Finally, after an oxidation process, under open air condition, the aromatic product **34** is formed (Scheme 12).

**Scheme 13:**
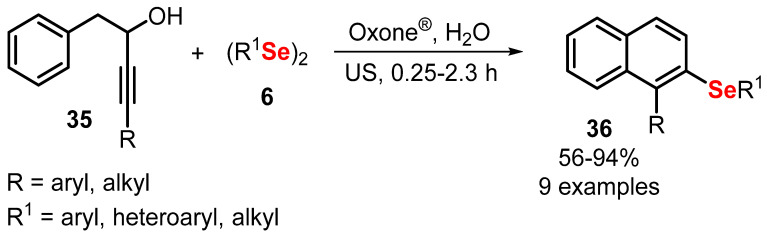
Ultrasound-mediated cyclization of alkynols **35**.

In 2018, Perin and co-workers described the ultrasound-mediated cyclization of alkynols **35**, promoted by the system (R^1^Se)_2_ **6**/Oxone^®^, to access 2-organoselenyl-naphthalenes **36** [38]. The method was suitable for electron-rich and electron-deficient alkynols, giving nine different 2-organoselanyl-naphthalenes **36** in moderate to excellent yields (Scheme 13).

**Scheme 14:**
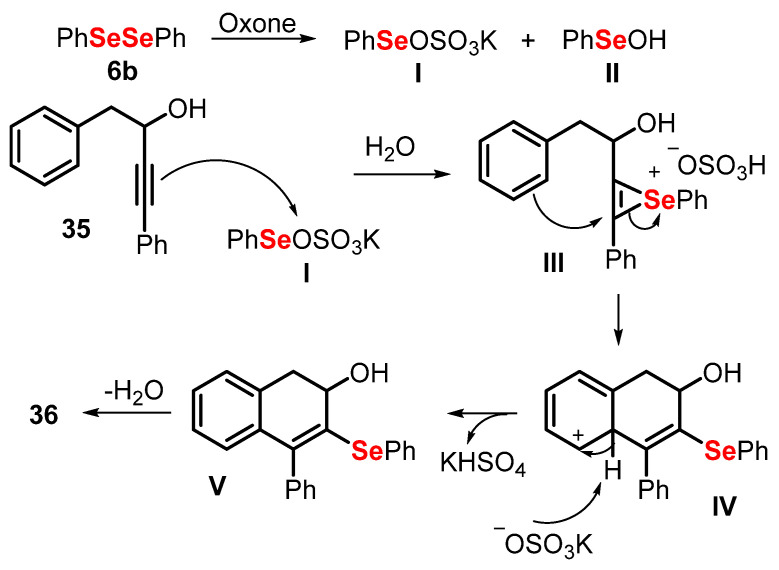
Mechanism of the US-mediated cyclization of alkynols **33**.

The proposed mechanism initially involves the Se-Se bond oxidation by Oxone^®^, affording the reactive intermediates **I** and **II**. Then, the Se-based electrophilic species **I** reacts with the C≡C bond of the alkynol **35**, to produce the seleniranium intermediate **III**, which undergoes an intramolecular 6-*endo*-*dig* cyclization to give the intermediate **IV**. Finally, a deprotonation to regenerate the aromaticity of the system affords the dihydronaphthalene **V**, which is converted to the desired product **36** through a dehydration process (Scheme 14).

**Scheme 15:**
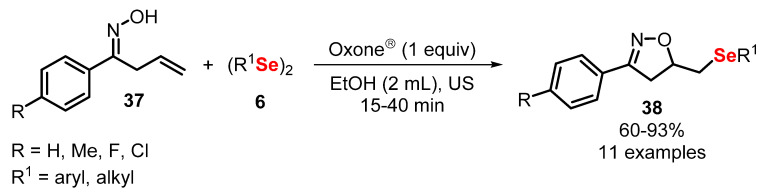
Selenocyclization of β,γ-unsaturated oximes **37**.

In 2020, Perin and co-workers also reported an ultrasound-assisted selenocyclization of β,γ-unsaturated oximes **37**, promoted by the system (R^1^Se)_2_ **6**/Oxone^®^, to access dihydroisoxazolines **38** [39]. The protocol has demonstrated to be very efficient, affording the desired products in moderate to excellent yields in just a few minutes under ultrasound irradiation (Scheme 15).

**Scheme 16:**
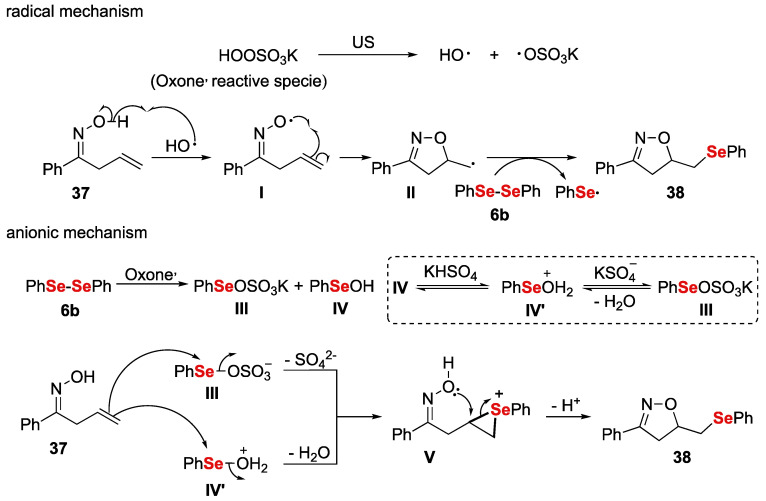
Mechanism of selenocyclization of β,γ-unsaturated oximes **37**.

After some control experiments, two possible mechanisms for the transformation were proposed, following a radical and an anionic pathway (Scheme 16). Regarding the radical pathway, initially the reactive peroxide species present in Oxone^®^ (HOOSO_3_K) undergoes an ultrasound-promoted homolytic cleavage, leading to the formation of a hydroxyl radical (HO^•^), which triggers a radical intramolecular cyclization of the oxime **37**, affording the cyclized alkyl radical **II**. Then, the species **II** reacts with the Se-Se bond, resulting in the desired selenyldihydroisoxazole **38**, and releases one equivalent of the Se-centered radical, which is eventually oxidized to diphenyl diselenide **6b** (Scheme 16, radical pathway). On the other hand, regarding the anionic process, from the reaction between Oxone^®^ and diphenyl diselenide **6b**, the intermediates **III** and **IV** are initially generated, among which the species **IV** is easily converted, in acidic medium, to the more electrophilic species **IV’**. In the sequence, the oxime **37** reacts with the electrophilic species **III** and **IV’**, to result in the seleniranium intermediate **V**. Finally, a dehydrogenative intramolecular annulation drives the reaction to the desired product **38** (Scheme 16).

**Scheme 17:**
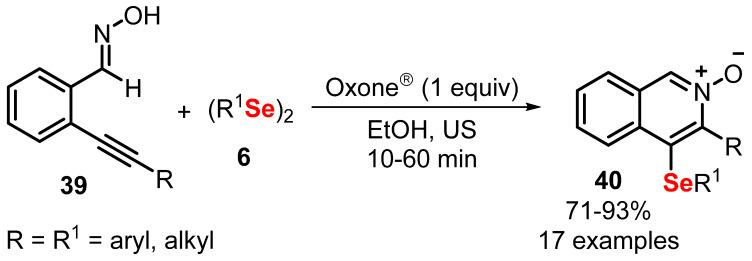
Electrophilic cyclization of alkynylbenzaldoximes **39**.

Employing the system (R^1^Se)_2_ **6**/Oxone^®^, Perin and co-workers have reported, in 2021, an electrophilic cyclization of alkynylbenzaldoximes **37** under ultrasound irradiation to construct 3-organyl-4-(organylselanyl)isoquinoline-2-oxides **38** [40]. The reactions were conducted in the presence of ethanol, and a wide range of substrates (electron-rich and electron-deficient) were satisfactorily employed, affording the desired products **38** in good to excellent yields, in just few minutes. It is worthy of mention that the standard reaction condition was highly scalable, up to ten times, providing the product **38** derivative in 85% yield (Scheme 17).

### 2.3. Electrochemically Promoted Reactions

Electrochemical organic synthesis has emerged as a powerful tool for obtaining several compounds, being an environmentally friendly synthetic strategy compared to the traditional methods [41,42,43], besides being industrially employed [44]. From the appropriate choice of electrodes and electrolytes, the electrosynthesis allows for control of selectivity without the use of external oxidants, catalysts and/or expensive and toxic ligands. By controlling some parameters, such as potential and current, it is possible to control the reaction rate; many reactions occur at room temperature and atmospheric pressure. In these reactions, the electric current promotes redox transformations via anodic oxidation and cathodic reduction without the presence of external agents, generating radical, cationic or anionic intermediates that can promote substitution, addition, elimination and even cyclization reactions.

The great advantages of electrosynthesis have also reached the chemistry of organochalcogens. In view of this, many research groups in recent years have been dedicated themselves to the chalcogenofunctionalization of organic molecules, mainly of biological interest, using this alternative approach [45].

**Scheme 18:**
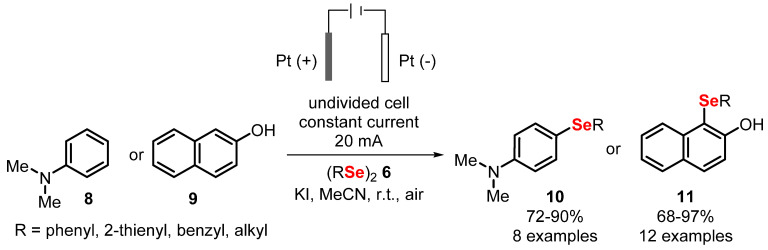
Selenylation of arenes **10** and **11**.

In 2019, Mendes and co-workers [46] described the electrochemical oxidative C(sp^2^)–H selenylation of *O*- and *N*-containing arenes **8** and **9** (Scheme 18). Through a regioselective methodology that employed Pt electrodes, KI as an electrolyte and using a half molar equivalent of diselenides **6,** it was possible to obtain a series of unsymmetrical selenides **10** and **11** in good to excellent yields. It is important to highlight that the developed protocol was carried out in an open atmosphere, at room temperature, with atomic economy and safe scalability.

**Scheme 19:**
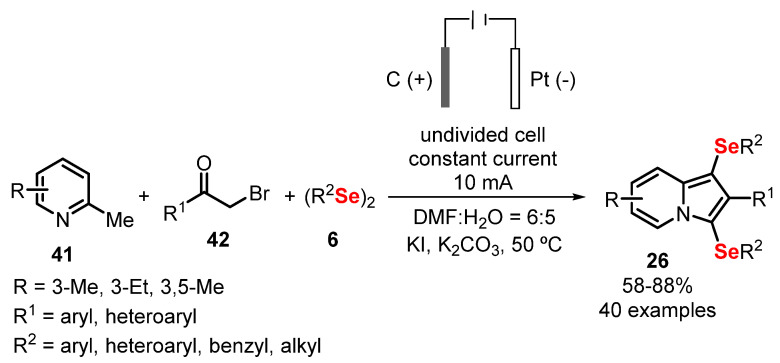
Multicomponent reaction via electrochemistry.

In the same year, Cao et al. [47] promoted the electrosynthesis of selenylated indolizines **26** through a multicomponent process, from 2-methylpyridines **41**, α-bromoketones **42** and diselenides **6** (Scheme 19). The preparation of the target molecules **26** was accomplished via a mixture of DMF:H_2_O (6:5) as solvent, K_2_CO_3_ as base, KI as electrolyte and an electric current of 10 mA, without the need for external oxidants and transition-metal catalysts. The developed green synthetic method provided 40 examples, with yields ranging from 54% to 88%.

**Scheme 20:**
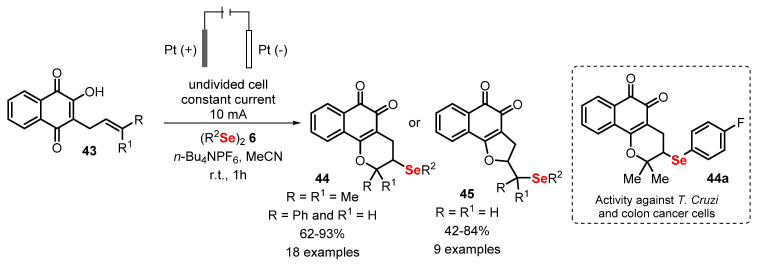
Electrochemical-mediated synthesis of selenium-containing naphthoquinones **44** and **45** with biological activity.

The electrochemical selenylation/cyclization of naphthoquinones was described by Da Silva Júnior and co-workers [48] as an efficient and reliable method for the achievement of molecules with great biological potential: **44** and **45** (Scheme 20). A wide range of selenium-containing multifunctional redox quinoidal compounds has been synthesized by using lapachol **43** or lawsone derivatives-in undivided electrochemical cells–diselenides **6** (1.0 equivalent), *n*Bu_4_NPF_6_ (1.0 equivalent) as electrolyte and acetonitrile as solvent. Moreover, the selenium-containing naphthoquinones **44** and **45**, were obtained in only one hour at room temperature, presenting biological activity against five cancer cell lines and Trypanosoma cruzi.

**Scheme 21:**
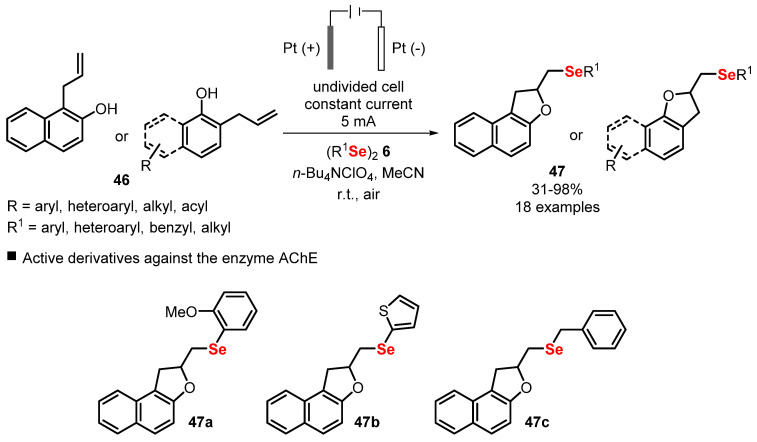
Preparation of selenyl-dihydrofurans **47** via intramolecular electrochemical oxidative oxyseleno-cyclization.

Another valuable approach to obtaining selenium-containing molecules with important pharmacological properties is the intramolecular electrochemical oxidative oxyseleno-cyclization of allylnaphthol and allylphenol **46** derivatives with diselenides **6** (Scheme 21) [49]. Under greener conditions, Braga and co-workers prepared selenyl-dihydrofurans **47** with 0.2 equivalent of *n*Bu_4_NClO_4_ as an electrolyte and MeCN as solvent within 2 h at room temperature. The desired products were synthesized in good to excellent yields and showed great potential against Alzheimer’s Disease.

**Scheme 22:**
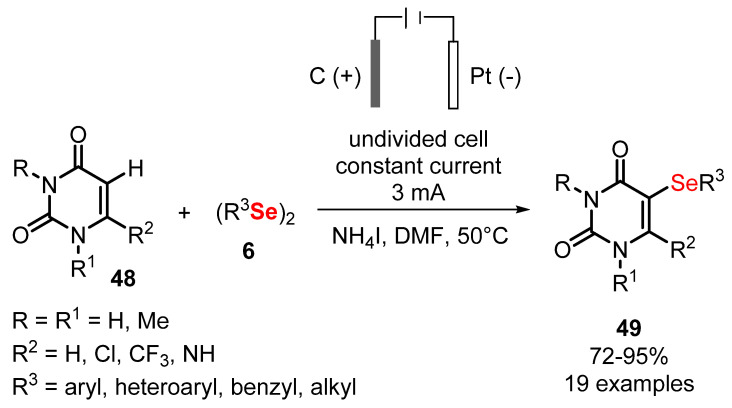
Protocol developed for obtaining 5-selenouracils **49**.

Uracil is a nitrogenous base present in our RNA and its selenylation as well as its derivatives **48** have been developed by Xu and co-workers [50] through the use of electrochemistry. The protocol established used diaryl diselenides **6**, valuable precursors of selenium-centered reagents [51], as selenylating agents in the presence of NH_4_I and DMF under air at 50 °C for 2 h, and allowed for the preparation of nineteen 5-selenouracils **49** (Scheme 22). In general, the yields were excellent, ranging from 72% to 95% in 3 mA of constant current, with C as anode and Pt as cathode [50].

**Scheme 23:**
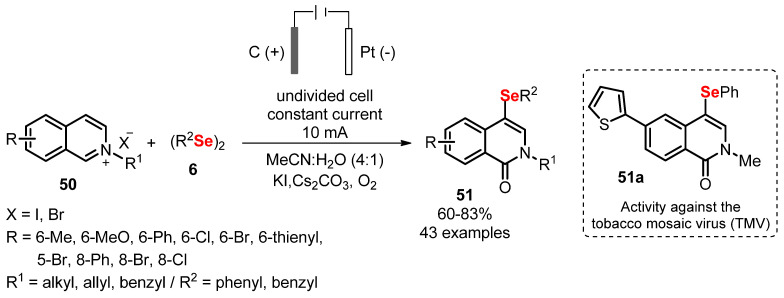
Regioselective selenylation/oxidation of *N*-alkylisoquinolinium salts **50** under electrolytic conditions.

As it is possible to observe, the methodologies involving electrosynthesis are environmentally friendly alternatives for the construction of C-heteroatom bonds and have attracted considerable attention. Following this premise, in 2020, Cao et al. [52] described an regioselective selenylation/oxidation of *N*-alkylisoquinolinium salts **50** via the C(sp^2^)−H bond functionalization with diselenides **6** (Scheme 23). In the presence of MeCN:H_2_O as solvent, KI as supporting electrolyte and Cs_2_CO_3_ as base, a series of selenylated isoquinolones **51** were easily accessed under undivided cell electrolytic conditions. In addition, the derivative **51a** exhibited potent antiviral activity.

**Scheme 24:**
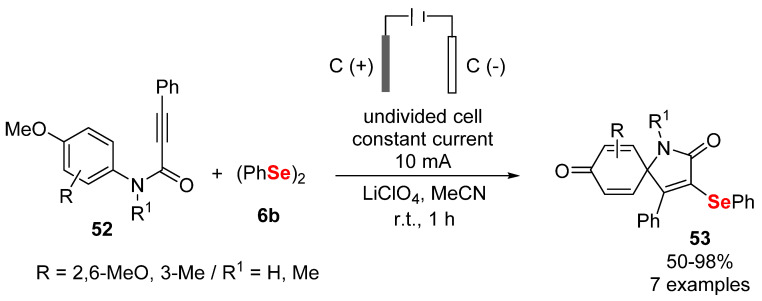
Electrosynthesis of selenylated spiro[4.5]trienones **53**.

Chen and co-workers [53] developed an interesting methodology for obtaining halogenated and selenylated spiro[4.5]trienones. The simple electrocatalytic reaction occurred without metal catalyst or oxidant, using the appropriate *N*-aryl alkynamides **52** and diphenyl diselenide **6b** (Scheme 24). The target selenylated spiro[4.5]trienones **53** was achieved in good to excellent yields under acetonitrile, at room temperature and in an undivided cell for 1 h.

**Scheme 25:**
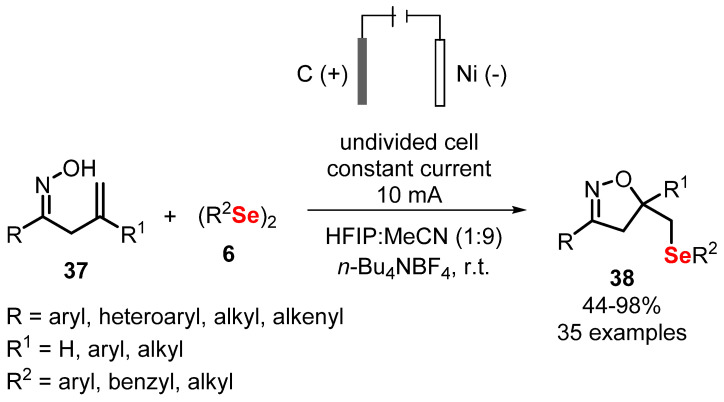
Electrochemical oxidative tandem cyclization for the synthesis of selenium-containing isoxazolines derivatives **38** with quaternary carbon center.

Recently, a methodology to prepare a series of isoxazoline derivatives **38** was described by Xu et al. [54] (Scheme 25). The intramolecular oxidative cyclization of β,γ-unsaturated oxime **37** with diselenides **6** has been carried out under mild reaction conditions in the absence of metals and oxidants, using CH_3_CN/HFIP (9:1) as solvent and *^n^*Bu_4_NBF_4_ as electrolyte. With the developed protocol, it was possible to access a series of 35 compounds **38** in up to 98% yield. In addition to being tolerable to a wide range of substrates, the protocol showed good results in scalability.

**Scheme 26:**
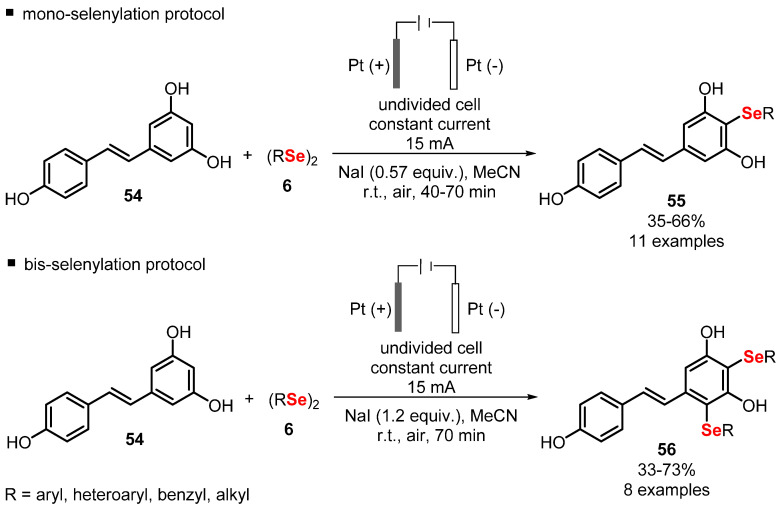
Electrochemical oxidative C(sp^2^)-H bond selenylation of resveratrol **54**.

Later on, Mendes and co-workers [55] described the functionalization of resveratrol **54** through an efficient environmentally benign procedure (Scheme 26). The unprecedented mono- and bis-selenylated compounds **55** and **56** were achieved in the absence of transition metal, oxidant and base, under air, using NaI as electrolyte and different diselenides **6** as a selenium source. The regioselective electrochemical oxidation employed resulted in the corresponding isolated selenylated products in moderate to good yields.

**Scheme 27:**
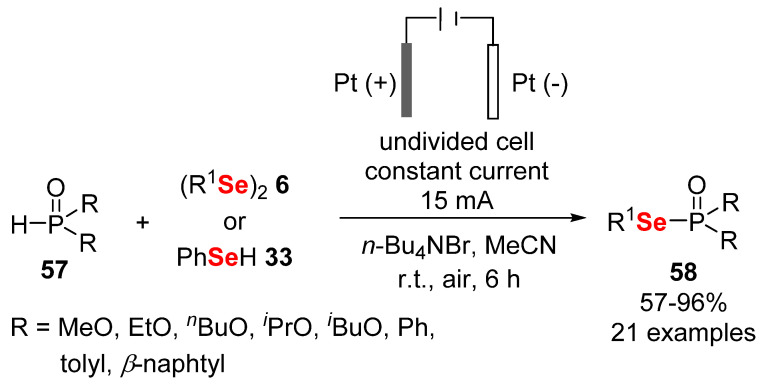
Electrochemical obtention of organoselenophosphorus **58** from phosphonates and phosphine oxides.

Organoselenophosphorus derivatives have been gaining attention due to application in several areas [56]. Among the methodologies developed for the synthesis of this type of molecule, the electrocatalytic-mediated selenylation of phosphonates or phosphine oxides **57**, by Cai et al. [57], is highlighted (Scheme 27). The ecofriendly protocol was conducted during 6 h under air and employed a platinum anode and a platinum cathode with constant current, nBu_4_NBr and acetonitrile. The authors showed the synthesis of 21 organoselenophosphorus **58** with yields up to 96%.

### 2.4. Photochemistry

The use of photons of light as an energy source in the synthesis of organic compounds has been established as a promising strategy due to the large number of benefits from synthetic and environmental points of view [58,59,60,61,62,63]. Such methodologies are generally based on the ability of some photocatalysts, usually organic dyes, to absorb energy and initiate radical processes in organic molecules. The development of this type of protocol is of great importance, since radical reactions can be considered as an alternative to processes that do not occur or occur in an unsatisfactory way through ionic mechanisms and/or induction via conventional thermal heating.

Photo-induced reactions, as written above, offer several advantages over other methodologies, complying with various principles of Green Chemistry. In this context, light as a primary energy source is abundant, safe, easy to handle and is a high energy-efficiency provider. In addition, the photocatalysts, when necessary, can generate excited states in low reactive molecules, which permits their functionalization in a mild way through safer synthetic routes with a consequent reduction in the formation of undesirable side-products [64,65,66,67]. The apparatus used for light irradiation is generally easily assembled and uses low-cost materials such as light-emitting diodes (LEDs) (green, white or blue) or commercial CFL lamps, eliminating the need for any photochemical equipment [68,69].

All these advantages have meant that in the last seven years there has been a significant increase in the number of works reporting the use of photochemistry in the seleno-functionalization of several classes of organic substrates [70]. For example, the carbon-selenium bond formation in heteroarene-like indoles [71,72], imidazo[1,2*a*]pyridines [73,74], coumarines [75], benzothiazoles [76] and pyridines [77]; selenylation of naphthols [78], terminal alkenes [79,80] and alkynes [81,82] and sp^3^ carbons [83,84]; and selenocyclization reactions have been reported [85,86]. In these protocols, in general, the substrate, the selenylating reagent and the photocatalyst are stimulated by visible light irradiation via single electron transfer (SET) reactions. The generated radical species follow in the mechanistic process depending on the reaction medium involved.

**Scheme 28:**
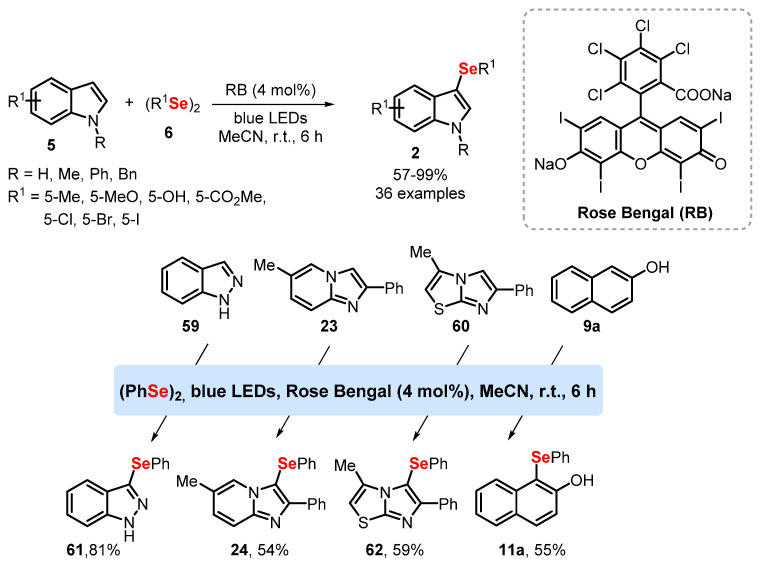
Rose Bengal-catalyzed synthesis of 3-selenylindoles **2**.

Among the developed methodologies, the photo-induced selenylation of several arene and heteroarene nuclei were developed by Braga and co-workers using diorganoyl diselenides **6**, the organic dye Rose Bengal as photocatalyst and blue LED as a light source (Scheme 28). This methodology proved to be efficient for the preparation of many biologically interesting selenium compounds, such as 3-selenylindoles **2**, which could be obtained with great structural diversity, in 6 h of reaction time and yields of up to 99%. Furthermore, this method allowed for C-Se bond formation in other nuclei, such as indazole **59**, imidazo[1,2-*a*]-pyridine **23**, imidazo[1,2-*b*]thiazole **60** and 2-naftol **9a [78]**.

**Scheme 29:**
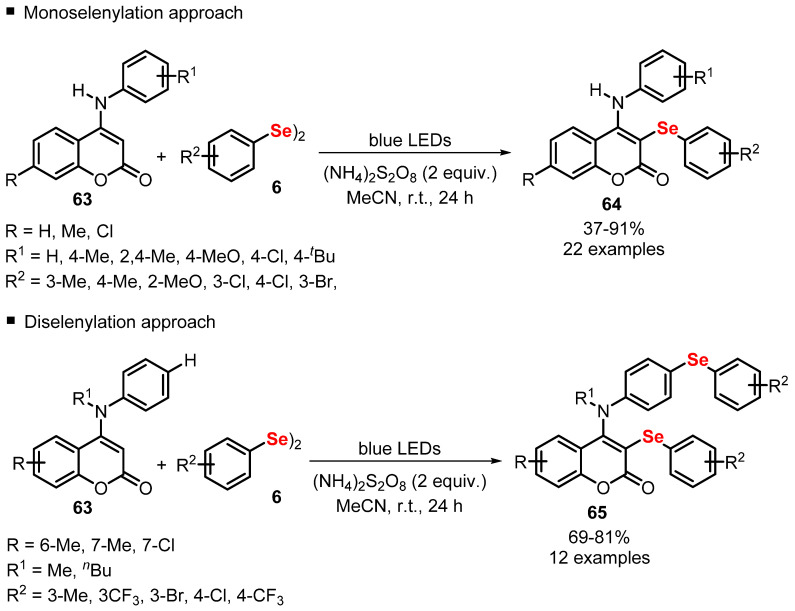
Selenylation of coumarins **63** promoted by blue LEDs.

In another approach, Yang and co-workers reported the photo-induced selenylation of 4-amino-substituted coumarines **63** using ammonium persulfate as a safe oxidant in the absence of photo-catalysts. The developed protocol used diaryl diselenides **6** as selenylating agents and allowed for the preparation of several mono- and bis-selenocoumarins **64** and **65** after 24 h of reaction at room temperature (Scheme 29). The yields were generally good, ranging from 37% to 91% [75].

**Scheme 30:**
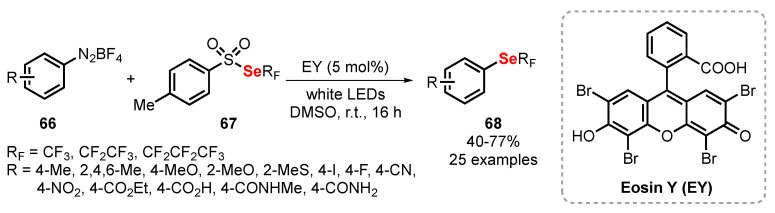
Perfluorometilselenylation of arenes **66** induced by white LED.

The introduction of a fluoroalkylselenyl motif into an organic molecule has been gaining prominence due to the various applications attributed to this class of compounds [87]. Among the methods already described, the fluoroalkylselenylation of arenes induced by white LED was developed by Tlili and co-workers in the reaction between arene diazonium salts **66** and trifluoromethyl tolueneselenosulfonate derivatives **67** (Scheme 30) [88]. The authors employed Eosin Y as photocatalyst, and the corresponding substitution products **68** were obtained in good yields at room temperature after 16 h.

**Scheme 31:**
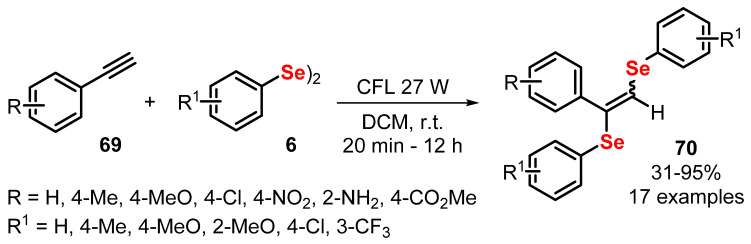
Photo-induced synthesis of 1,2-bis-organoylselenyl alkenes **69**.

Schneider and co-workers [89] reported a photo-induced process for the seleno-functionalization of terminal alkynes **69** (Scheme 31). By using diaryl diselenides **6**, the corresponding 1,2-bis-organoylselenyl alkenes **70** were obtained at room temperature in up to 12 h of reaction, in the absence of a photocatalyst. Moreover, the alkenes were prepared in good to excellent yields and regioselectivity, with preference for the formation of the *E* regioisomer.

**Scheme 32:**
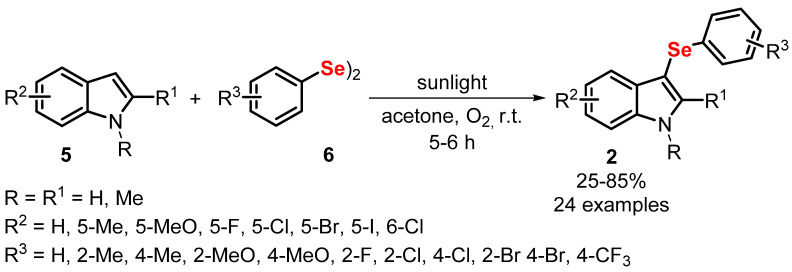
Sunlight-driven selenylation of indoles **5**.

Later on, the sunlight-driven direct selenylation of indoles **5** with diaryl diselenides **6** was reported by Kumar and co-workers [90]. The preparation of 3-selenylindoles **2** occurred in the presence of O_2_ as a benign oxidant, with acetone as solvent, at room temperature without photocatalysts or any other reagents, in up to 85% yield (Scheme 32). The use of sunlight is highly desirable as it is an abundant source of energy and allowed the preparation of sulfenyl- and tellurenylindoles in good yields, in times that ranged from 5 to 6 h.

**Scheme 33:**
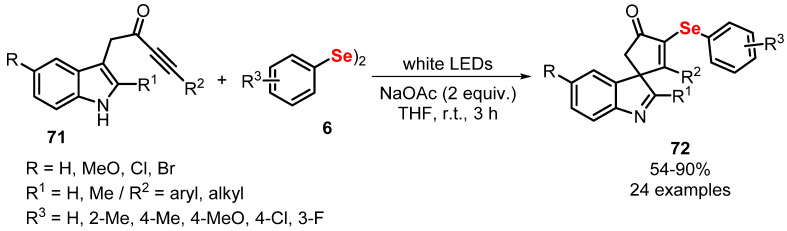
Synthesis of 3-selenospiroindolenines **72** promoted by white LEDs.

Visible light has also been successfully used to promote seleno-cyclization reactions via cascade processes (Scheme 33). In this context, Xu and co-workers promoted the spirocyclization of indolyl-ynones **71** with diaryl diselenides **6** in order to obtain 3-selenospiroindolenines **72**, which are attractive molecules from a biological point of view. The desired products were synthesized in up to 90% yield by using white LEDs, sodium acetate and THF as solvent, at room temperature. This method also presented good tolerance of the presence of different functional groups attached to the substrates, and the use of photocatalyst was not required [86].

**Scheme 34:**
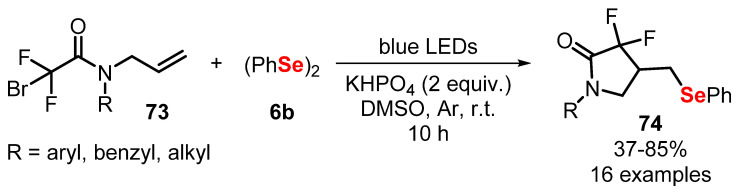
Blue LED-mediated cyclization of **73**.

Recently, the five-*exo*-*trig* cyclization of *N*-allylbromodifluoroacetamide **73** with diphenyl diselenide **6b** was performed through a photochemical process (Scheme 34). The corresponding products, 3,3-difluoro-β-lactams 4-seleno-substituted **74,** were obtained in moderate to good yields, mediated by blue LEDs and KHPO_4_. The developed protocol occurred at room temperature in 10 h without photocatalyst [91].

**Scheme 35:**
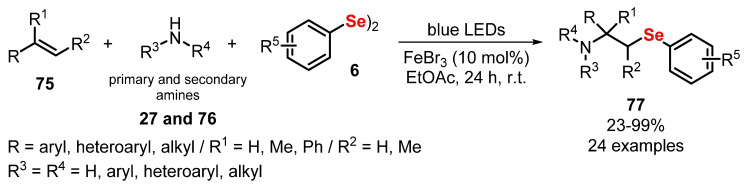
Visible light-induced iron catalyzed three-component reaction.

Later, Xia and co-workers reported the use of blue LEDs in the selenoamination of alkenes catalyzed by FeBr_3_. The developed methodology provided the synthesis of a series of β-aminoselenides **77** through a three-component reaction between alkenes **75**, amines **27** and **76** and diorganoyl diselenides **6** (Scheme 35). The desired products **77** were achieved in up to 99% yield at room temperature after 24 h, and the method proved to be efficient, with a large structural variation in the substrates [80].

**Scheme 36:**
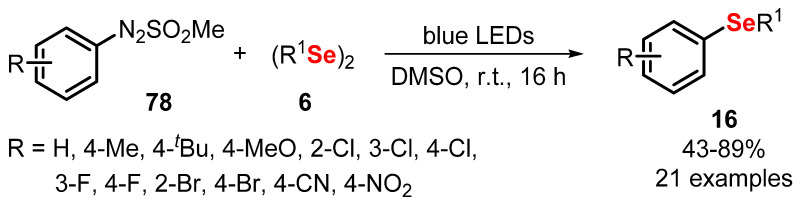
Photochemical synthesis of diorganyl selenides **16**.

The blue LED irradiation was also recently employed in the coupling reaction of aryl diazosulfones **78** and diorganyl diselenides **6** under a photocatalyst-free condition (Scheme 36). In the presence of DMSO as solvent, unsymmetrical diorganyl selenides **16** were obtained in yields ranging from 43% to 89% after 16 h [92].

**Scheme 37:**
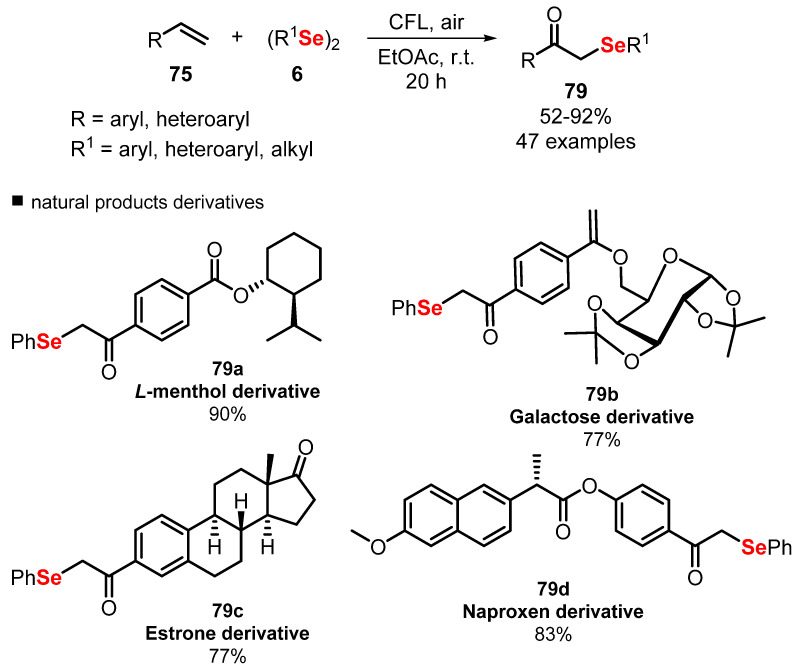
Visible light-driven selenylation of alkenes **75** and its application to biologically relevant molecules **79a**–**d**.

In 2021, an environmental process for the selenylation of alkenes **75** with diorganyl diselenides **6** was described by Liu and co-workers for the synthesis of α-alkyl selenomethyl ketones **79** (Scheme 37) [93]. The visible light-promoted protocol allowed for the efficient synthesis of the target molecules in 20 h using ethyl acetate as solvent, under air, at room temperature, avoiding the use of bases, oxidants or photocatalysts. Furthermore, the authors applied the same method to the introduction of the selenium moiety into biologically important molecules, such as L-menthol, galactose, the hormone estrone and the anti-inflammatory naproxen. The corresponding selenylated products **79a**–**d** were obtained in 77–90% yields.

**Scheme 38:**
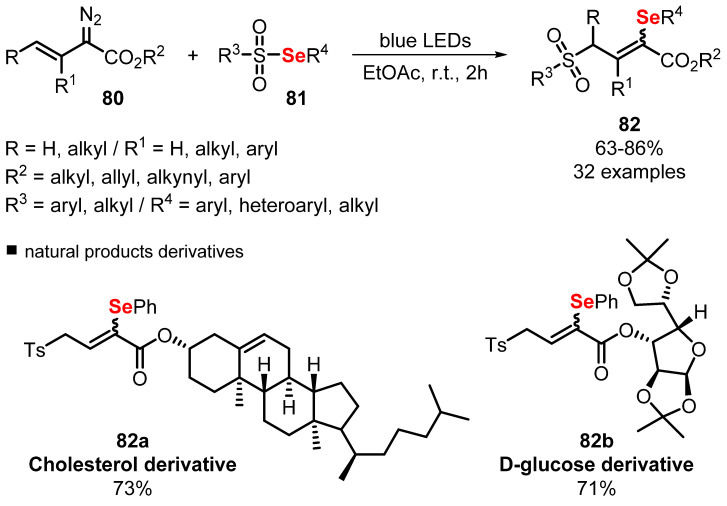
Blue LED-driven 1,3-selenosulfonates **82**.

In the same year, the photo-induced 1,3-addition of selenosulfonates **81** to vinyldiazo compounds **80** was reported by Zhou and Li (Scheme 38) [94]. The corresponding addition products **82** were prepared in the presence of ethyl acetate at room temperature with blue LED irradiation as a light source. The features of the protocol were the absence of photocatalysts, bases and oxidants, reactions carried out in open flask and high tolerance of functional groups. Furthermore, this methodology was applied in the functionalization of biomolecules such as cholesterol and D-glucose derivatives.

### 2.5. Mechanochemistry

Mechanochemical processes deal with an important premise of Green Chemistry, which is the promotion of reactions, whenever possible, under solvent-free conditions and short reaction times. The main advantages are the remarkable reduction in cost and energy demands involved in solvent production and purification. In addition, solvent waste generation is minimized, protecting the environment and reducing costs in recycling processes [95,96]. In this context, during the last decade, advances were achieved in the synthesis of organoselenium compounds under solvent-free, mechano-assisted conditions, opening up new horizons for the synthetic technologies of these compounds.

**Scheme 39:**
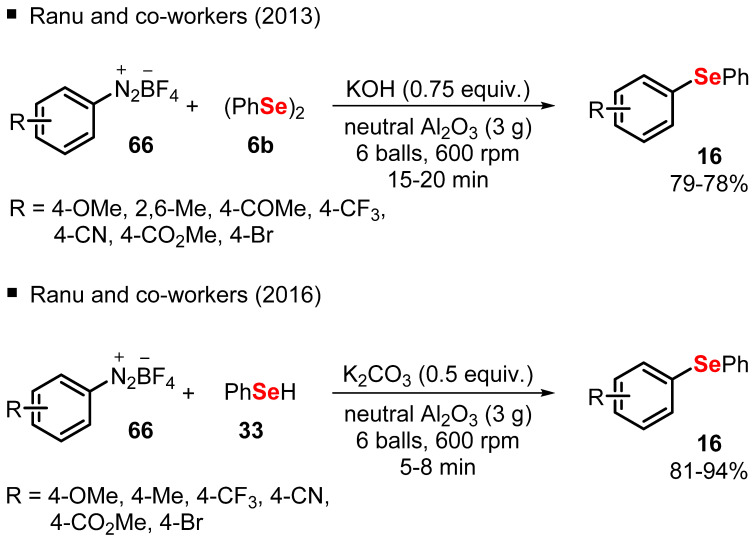
Synthesis of unsymmetrical diaryl selenides **16**.

In 2013, Ranu and co-workers disclosed the first synthesis of unsymmetrical diaryl selenides **16** under ball-milling conditions [97]. This approach involves the reaction between several aryl diazonium tetrafluoroborates **66** and diphenyl diselenide **6b** in the presence of Al_2_O_3_ and KOH in a reactor bearing six balls operating at 600 rpm, in the absence of TM-catalysts and solvent. Satisfactorily, eight unsymmetrical selenides **16** were obtained in good yields after a few minutes under milling, presenting an excellent tolerance of electron-rich and electron-deficient aryl diazonium tetrafluoroborates **66** (Scheme 39). It is worthy of mention that the protocol was also employed with diphenyl disulfides and ditellurides.

Later, in 2016, Ranu and co-workers also reported a very similar method to prepare unsymmetrical diaryl selenides **6**, this time employing selenols **33** as the selenium source [98]. The reactions were conducted in the presence of Al_2_O_3_ and K_2_CO_3_ instead of KOH, also in a reactor bearing six balls, operating at 600 rpm. Satisfactorily, shorter reaction times were needed to afford the products **16** in good to excellent yields. The processes were conducted very well, presenting an excellent tolerance to different electron-rich and electron-deficient substrates, including strong electron-withdrawing groups (CF_3_ and CN). It is worthy of mention that thiols were also employed as substrates to afford the respective unsymmetrical diaryl sulfides (Scheme 39).

**Scheme 40:**
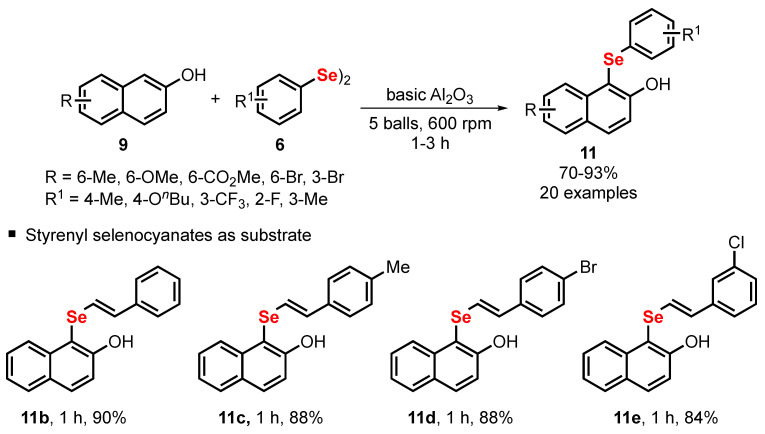
Selenylation of 2-naphtols **9**.

Very recently, in 2020, Ranu and co-workers reported a mechanochemically mediated protocol for the selenylation of 2-naphthols **9**, employing diaryl diselenides **6** as the selenylating agent in the absence of TM-catalyst, oxidants and solvent [99]. The protocol was conducted in a five-ball reactor, operating at 600 rpm for 1 h, in the presence of basic Al_2_O_3_ (washed with a 15% KOH solution and dried). Under the optimized reaction condition, twenty 1-selenyl-2-naphthols **11** were prepared in good to excellent yields, presenting an excellent tolerance to several electron-rich and electron-deficient 2-naphthols and diaryl diselenides (Scheme 40).

Additionally, the protocol applicability was evaluated, and styryl selenocyanates, phenylselenyl chloride/bromide and cyanide were also employed as substrates. Satisfactorily, the products **11b**–**e** were obtained in very good to excellent yields after 1 h under milling condition (Scheme 40).

**Scheme 41:**
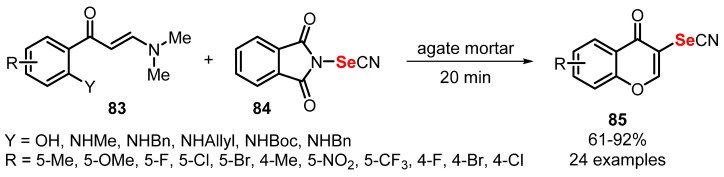
Electrophilic cyclization of 2-substituted enaminones **83**.

More recently, Xiao, Ren and co-workers reported a grinding-mediated electrophilic cyclization of 2-substituted enaminones **83** in the presence of *N*-selenocyanophthalimide **84** as a selenocyanating reagent to afford 3-selenocianato-substituted chromones **85** [100]. The reactions were conducted in an agate mortar in the absence of solvent, and after grinding for just 20 min at room temperature, the desired products **85** were obtained in moderate to excellent yields, presenting a good substrate tolerance. It is worthy of mention that the protocol was highly scalable to gram-scale, resulting in the neutral derivative **85** in 71% yield (Scheme 41).

### 2.6. Flow Chemistry

Flow chemistry is an innovative technology in which chemical reactions are conducted in a continuously flowing stream and has received considerable attention from the chemical community in recent years. Both academia and industries, especially pharmaceutical ones, have shown this interest due to the great advantages presented by this technique, such as: broad versatility in applications and excellent reproducibility and safety, among others [101]. Reactions conducted under these conditions have emerged as environmentally friendly alternatives for synthesizing products of interest faster and more safely than those reactions performed on benches. Moreover, through this technological platform, it is possible to carry out difficult or even impossible reactions to perform in batch mode [102].

Due to these characteristics, flow chemistry is inspiring the development of green [103,104,105] and modern methods [106,107,108,109,110,111,112,113,114,115], and many applications in biocatalysis, photochemistry and electrochemistry have been described so far [116,117,118]. However, involving chalcogens, especially selenium, in this chemistry is quite recent. The use of selenium compounds as catalysts, as well as their incorporation into other organic molecules, have already been developed through flow chemistry and are described below.

**Scheme 42:**
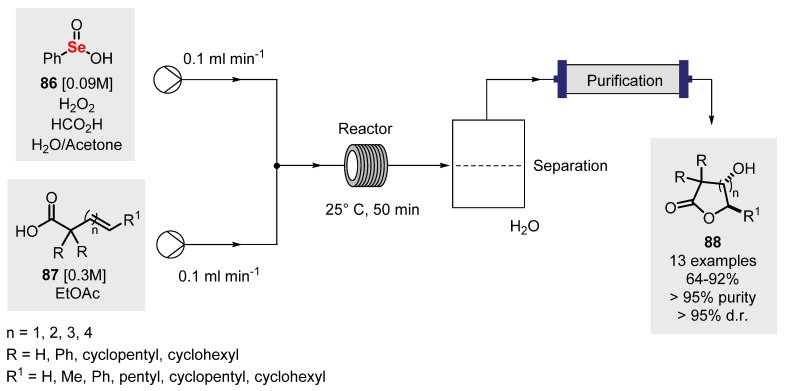
General flow system for the synthesis of hydroxy lactones **88**.

In 2017 [119], the first study using a selenium-mediated catalysis conducted under flow conditions (Scheme 42) was reported. Lactone rings and their precursors are often found in natural and bioactive compounds. As a result, their synthesis is an important area to be explored, and in this work the authors promoted the efficient and environmentally adequate synthesis of hydroxy lactones **88**. This methodology is based on the oxidation of functionalized alkenoic acids **87** followed by their internal cyclization using PhSeO_2_H **86** as the pre-catalyst to form in situ PhSeO_3_H via reaction with an oxidant. The reactants were mixed at a flow rate of 0.1 mL.min^−1^ at 25 °C, and the desired lactones were efficiently obtained after automated purification within 50 min. This method allowed for the obtention of 13 examples with high purity, excellent diastereomeric ratio and high yields, after the introduction of an online continuous system of purification.

**Scheme 43:**
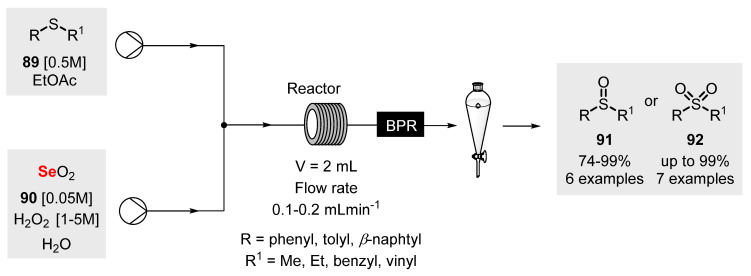
Flow process to obtaining sulfoxides **91** and sulfones **92** from different sulfides **89**.

In the recent past, following the idea of applying selenium-containing catalyst in a continuous flow system, Santi et al. [120] promoted the synthesis of sulfoxides **91** and sulfones **92** through a bioinspired approach (Scheme 43). In this process, SeO_2_ **90** was oxidized to perselenic acid; adeguately tuning the amount of H_2_O_2_ provided the products of interest in isolated yields ranging from 74% to >99%, by reacting with a wide range of sulfides **89**. Scalability studies of the reaction, under flow conditions, were also promising, showing the efficacy, simplicity, selectivity and eco-sustainability of the proposed protocol.

**Scheme 44:**
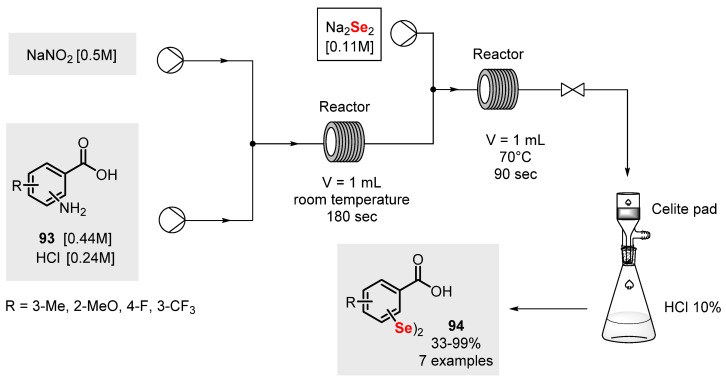
Continuous flow two-step synthesis of 2,2′-diselenobis(benzoic acid) derivatives **94**.

From a green chemistry perspective, continuous flow technology has many advantages over traditional batch approaches. In this context, Sancineto and co-workers [121] performed the first synthesis of the key building block 2,2′-diselenobis(benzoic acid) derivatives **94**, under flow conditions (Scheme 44). The formation of the target molecules took place in a reactor, heated to 70 °C for 90 s, through the mixture of a diazonium salt solution of anthranilic acid derivative **93** and a Na_2_Se_2_ solution. A diversity of diselenides was possible to be obtained, with good to excellent yields, demonstrating a great advance in the reduction of time and increase in the production of this class of compounds.

**Scheme 45:**
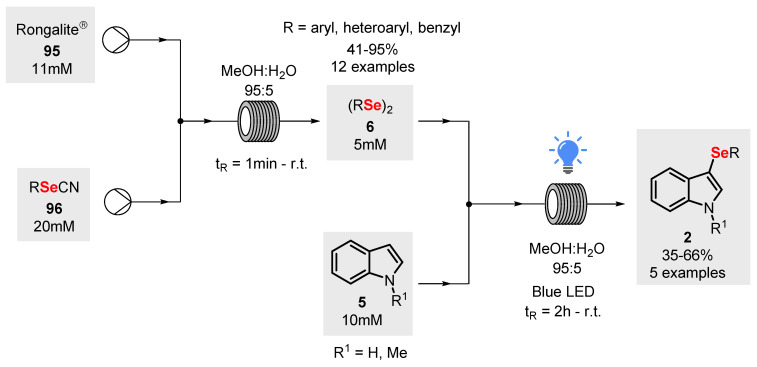
Selenylation of electron-rich arenes **5** through flow-chemistry protocol.

Flow chemistry applied to multistep reactions could be a promising platform to apply in medicinal, agrochemical and material sciences [122,123,124]. In this way, Oksdath-Mansilla group invested its efforts in the development of a continuous flow protocol for the synthesis of valuable selenides **2** from the combination of a chemical reduction and a photochemical C(sp2)−H activation reaction (Scheme 45) [125]. The first step consisted of the reduction of several selenocyanates **96** with Rongalite^®^ **95** to produce the corresponding diselenides in a perfluoroalkoxy (PFA) coil reactor. The yields of this step were between 41% and 95%, and it was possible to synthesize 12 different diselenides **6**. Second, the selenylation of electron-rich arenes **5** occurred through photoactivation of the Se-Se bond with blue LEDs in a mesoscale photochemical flow reactor. Products **2** were obtained in up to 66% yield over 2 h.

**Scheme 46:**
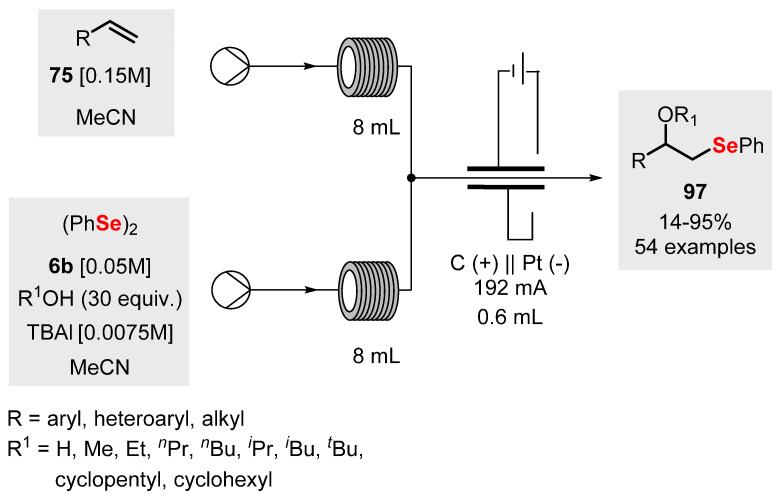
Representation of automated flow electrochemical selenenylation of alkenes **75**.

In 2020, an interesting combination of electro- and flow chemistry to obtain organoselenium compounds was described by Wirth and Amri [126]. This study was performed in an undivided cell using a Vapourtec automated flow system with an integrated ion electrochemical microflow reactor (Scheme 46). A library with 54 products of the selenenylation of alkenes **97** was obtained in yields of 14% to 95% in a fully autonomous way. Diphenyl diselenide **6b** was used in the reaction with different alkenes **75** in the presence of nucleophiles using a graphite electrode as the anode, a platinum electrode as the cathode and Bu_4_NI as electrolyte.

**Scheme 47:**
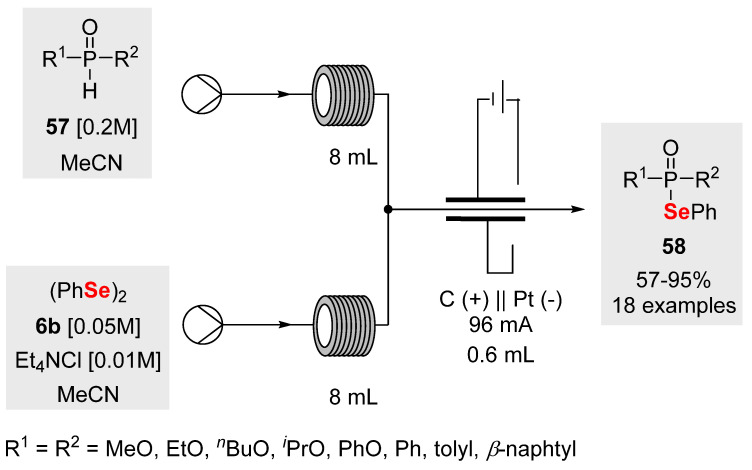
Representation of automated flow electrochemical synthesis of selenophosphites **58**.

Later, the same group [127] applied a similar approach to synthesizing chalcogenophosphites **58** in a fast, inexpensive, metal-free, environmentally friendly and automated way (Scheme 47). There are many bioactive molecules with chalcogen-phosphorus bonds, and preparing them sustainably and efficiently is a challenge for synthetic organic chemists. The reaction occurred between diphenyl diselenide **6b** and phosphite **57** in acetonitrile, with Et_4_NCl as electrolyte at 25 °C in the same electrochemical flow reactor described in Scheme 47. With this continuous flow protocol, it was possible to prepare 18 selenophosphites **58** in good to excellent yields in short reaction times.

**Scheme 48:**
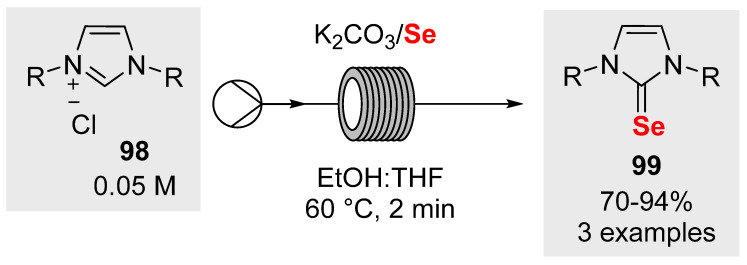
Synthesis of selenoureas **99** using a continuous flow system.

In 2021, Nolan and colleagues [128] reported the first synthesis of selenoureas using a continuous flow system. Unlike their sulfur isosteres, selenium-*N*-heterocyclic carbene compounds **99** were obtained using a heterogeneous Se/K_2_CO_3_ microreactor (Scheme 48). From different ligands **98**, it was possible to synthesize three selenoureas, even on a large scale, in only 2 min. This was considered to be a great advance for obtaining these species, which are important in coordination chemistry.

## 3. Application of Selenium or Organoselenium Compounds as Catalysts in Oxidation Reactions

**Figure 2:**
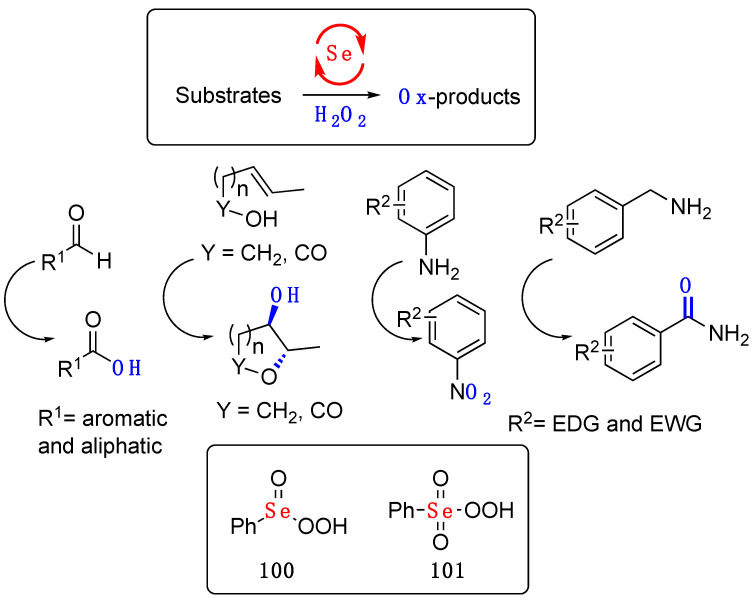
Se-catalyzed bioinspired oxidative transformations.

The use of organoselenium compounds as bioinspired catalyst for chemical transformations can be traced back to 1984, when Sies demonstrated that ebselen was able to mimic the activity of the key antioxidant enzyme glutathione peroxidase (GPx). This selenoenzyme removes harmful peroxides (H_2_O_2_ or organic peroxides) from the cell using them to convert glutathione (GSH) into the corresponding disulfide and leading to the formation of harmless molecules, such as water or alcohols [129,130,131]. After this milestone, several research groups focused their attention on the preparation of small molecules acting as GPx-mimics to be exploited for pharmacological or synthetic organic chemistry purposes [4,132,133]. In this latter research field, the possibility of using a selenium-based bioinspired catalyst to activate hydrogen peroxide, has permitted the development of oxidative protocols flexible enough to allow for transformations of a wide range of substrates, while addressing Green Chemistry principles (Figure 2).

Some of us have contributed to this specific research field with a couple of significant reports. In 2015, a phenylseleninic acid-catalyzed protocol for the conversion of aromatic and aliphatic aldehydes into their corresponding carboxylic acids, in the presence of one equivalent of a 10% (*w*/*w*) solution of hydrogen peroxide at room temperature, was developed. Such a method permits the gram scale preparation of benzoic acid by recovering the catalytic system several times. In addition, by carrying out the reaction in the presence of alcohols, it was possible to convert aldehydes into esters in a single step [134].

Later on, the bioinspired oxidation method was applied to the cyclofunctionalization of olefines bearing an internal nucleophile. In particular, alkenoic acids, as well as alkenols, proved to be suitable substrates for the preparation of hydroxy lactones or cyclic ethers in excellent yields and *trans* diasteroselectivity. The reaction took place in water as solvent, in the presence of 5 mol% of the catalyst and 4 equivalents of H_2_O_2_. An enantioselective approach was also attempted by using a chiral diselenide as pre-catalyst, allowing for the obtention of the target compound with a moderate enantioselectivity [119,135].

Very recently, Tanini and co-workers reported the conversion of aromatic primary amines into nitro compounds by using 20 mol% of the pre-catalyst, diphenyl diselenide. Aliphatic amines were unreactive, but when benzylamines were used as substrates, benzenamides were obtained in moderate yields [136]. From a mechanistic point of view, the conversion of aldehydes into carboxylic acids and esters and the preparation of nitro compounds from amines have in common the actual catalyst, which is phenylseleninic acid. It yields the oxygen-transfer species, phenylperseleninic acid (compound **100**, Figure 2), when it reacts with hydrogen peroxide [134,136]. In the case of the oxidation of carbon-carbon double bonds and the successive cyclofunctionalization reaction, the actual catalyst should be perselenonic acid (compound **101**, Figure 2), as demonstrated by Back very recently [137].

**Scheme 49:**
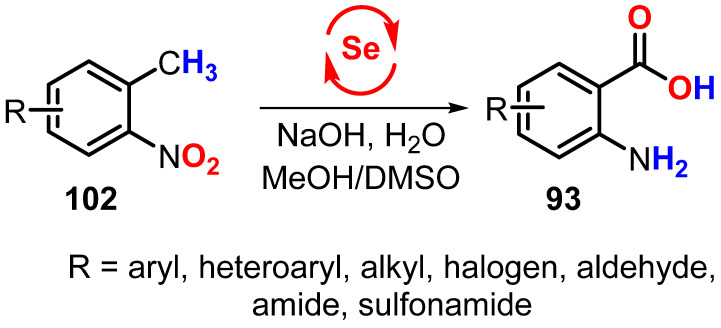
Conversion of nitrotoluenes **102** into anthranilic acids **93**.

A last example of an Se-catalyzed oxygen transfer reaction was reported by Jiang et al. in 2021. The use of elemental selenium (30 mol%) in a basic MeOH/DMSO mixture at 90 °C permitted the conversion of *o*-nitrotoluenes (**102**) into anthranilic acids (**93**) in good to excellent yields. This protocol is atom- and redox-economic, since no external oxidant is added to the reaction mixture (Scheme 49) [138].

## 4. Organoselenium Chemistry in Non-Conventional Solvents

One of the most important Green Chemistry principles is the one in which the use of safer solvents and auxiliaries is suggested. Water fully embodies the feature of green solvent since it is completely safe for the operator and, if recycled and reused, it can also be considered safe for the environment compared to organic solvents.

In recent years, several examples of organoselenium chemistry performed in water have been reported [139,140]. Here, just the most significant examples will be cited.

**Scheme 50:**
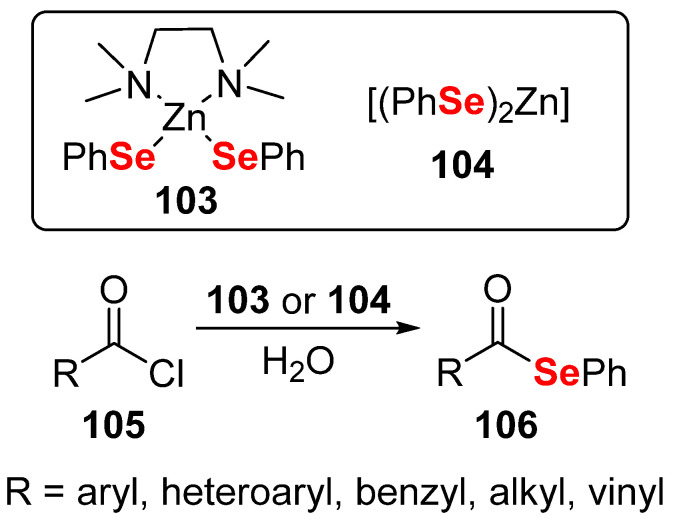
Preparation of selenoesters **106**.

Selenoesters (**106**) are valuable organic frameworks having a wide range of applications, such as in the preparation of liquid crystals [141] or pharmacologically active compounds [142,143], besides their importance in synthetic organic chemistry as selenols protecting groups or acyl transfer reagents [144,145]. In 2017, some of us reported the synthesis of selenoesters starting from two isolable Se/Zn complexes in reactions with acyl chlorides (Scheme 50) [146]. Zinc phenylselenolates are the only class of bench-stable, nucleophilic selenium species that are easy to prepare and handle [147]. The reagents reported in Scheme 2 represent a more atom-economic version of PhSeZn-halides, that proved to be highly efficient selenylating agents under “on water” [148] and solvent-free conditions [149]. By using **104**, higher yields of aromatic selenoesters were obtained, while **103** was suitable in the preparation of aliphatic ones. The complex **103** proved to be amenable to dispersion onto a solid support for the continuous synthesis and purification of selenoesters in an automated fashion [146]. Interestingly, by using **104** and carrying out the reaction in THF, selenoesters are not formed; instead, the solvent ring-opening is observed [150].

**Scheme 51:**
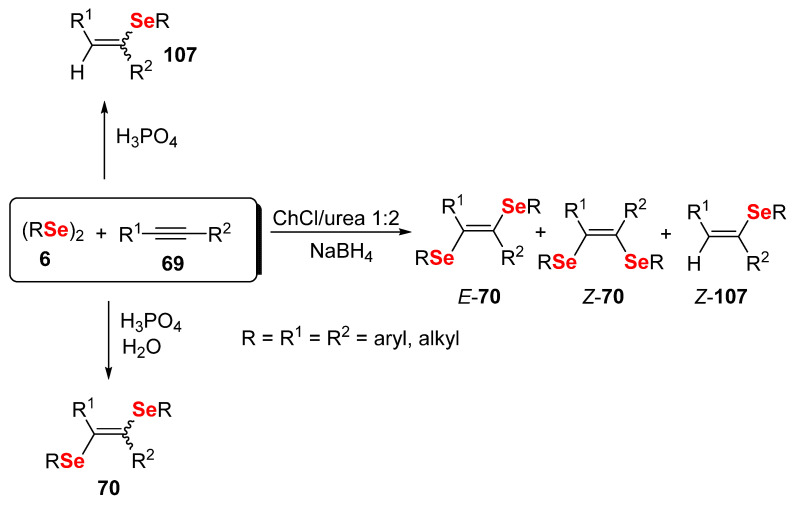
Preparation of vinyl selenides using DES.

Vinyl selenides are valuable tools in organic chemistry [151,152,153] and examples of their synthesis in unconventional solvents have been reported recently. Lenardão and co-workers reported the preparation of these kinds of compounds through the hydrochalcogenation of alkynes in deep eutectic solvents (DES) as reaction media, and in particular, a 1:2 mixture of choline chloride (ChCl) and urea was used (Scheme 51) [154].

With this method, the authors were able to flexibly obtain an array of densely functionalized vinylselenides (compounds **70** and **107**) in yields ranging from fair to excellent, despite mixtures of *E*, *Z*, mono- and bis-selenylated compounds being obtained, mainly on the basis of whether the alkyne substrate was a terminal or internal one [154]. The same research group demonstrated that selectivity can be tuned toward the monoselenylated compounds, mostly as *E*-isomers, by running the reaction in a 50% wt solution of H_3_PO_2_ in THF, or toward the bis-substituted derivatives by removing the organic solvent [155] or using PEG-400 [156].

**Scheme 52:**
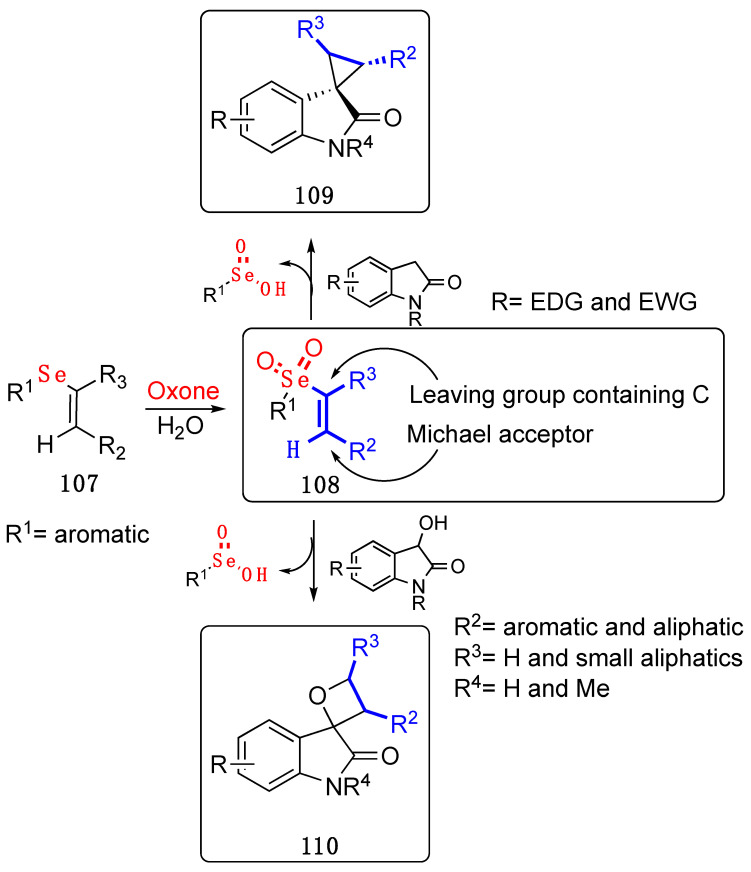
Preparation of vinyl selenones and their use as double electrophilic species.

Vinyl selenides are close precursors of vinyl selenones **108**, which are versatile building blocks, especially in C-C bond-forming reactions, due to their double electrophilic nature [157]. In 2018, Marini et al. developed an Oxone-based oxidation method to perform the synthesis of these scaffolds in water [158]. The versatility of vinyl selenones **108** has been demonstrated through their use as reagents for the synthesis of spirocompounds. For instance, spirocyclopropyl oxindoles (**109**) endowed with anti-HIV activity were prepared by treating vinyl selenones with variously decorated oxyndoles in aqueous basic conditions [159]. Similarly, the replacement of oxyndoles with 3-hydroxyoxindoles cleanly led to spirooxindole oxetanes (**110**) through a domino reaction (Scheme 52) [160].

## 5. Conclusions

Since its emergence in the 1990s, Green Chemistry has acquired a leading role in decision making in the fine chemical and pharmaceutical sectors, among other sectors of the chemical industry. As demonstrated in this updating review, a number of new strategies to prepare organoselenium compounds or the use of organoselenium derivatives in organic synthesis, both in catalysis and as building blocks, have appeared in the literature in the last eight years The ecofriendly methods for heterocycle preparation is just mentioned here because they have been fully covered in a recent review article [161]. The use of alternative energy sources, including mechanochemical, sonochemical and microwave has continually evolved in parallel with the availability of new, more accessible equipment. Flow chemistry, photochemistry and electrochemistry, in turn, appear as alternative allies to Green Chemistry that seem to have their place reserved in the chemistry of organoselenium compounds, as discussed in this review. The use of alternative solvents or even the solvent-free or solvent-minimized protocols also have a crucial role in achieving a more sustainable chemistry. In this review, it can be observed that several research groups around the world are working to make organoselenium chemistry green. Despite the path already covered and the frontiers pioneered in this field, there is still a long way to go before the practice of a more sustainable chemistry becomes routine in organic synthesis laboratories. Here, some important recent advances were presented, aiming to stimulate interest in the exploration of the “green side of the Moon”.
